# Computational simulation of the optical performance of an extended depth of focus intraocular lens in post-LASIK eyes

**DOI:** 10.1097/j.jcrs.0000000000001260

**Published:** 2023-11-01

**Authors:** Carmen M Lago, Alberto de Castro, Susana Marcos

**Affiliations:** 1. Instituto de Óptica, Consejo Superior de Investigaciones Científicas, Serrano 121, Madrid 28006, Spain; 2. 2Eyes Vision SL, Edison 3, E-28006 Madrid, Spain; 3. Center for Visual Sciences; The Institute of Optics and Flaum Eye Institute, University of Rochester, New York

## Abstract

**Purpose:**

To evaluate computationally the optical performance of Acrysof IQ Vivity Extended Depth of Focus (EDOF) Intraocular Lens (IOL) in post-LASIK eyes.

**Setting:**

Visual Optics and Biophotonics Laboratory, Madrid, Spain.

**Design:**

Experimental study.

**Methods:**

Computer pseudophakic eye models were implemented using reported post-LASIK corneal aberrations (refractive corrections from −7.5 to +4.5 D) and virtually implanted with monofocal (Acrysof IQ) or EDOF (Acrysof IQ Vivity) IOLs. Retinal image quality was quantified through Visual Strehl (VS). Depth-of-focus (DOF) was calculated from the through-focus VS curves. Halos were estimated from the light spread in the image of a pinhole. Those quantitative parameters were obtained for 5.0 and 3.0-mm pupil diameters.

**Results:**

Simulated virgin eyes showed VS of 0.89/0.99 with monofocal IOL and 0.74/0.52 EDOF IOL, for 5.0/3.0-mm pupils at best focus. VS decreased with induced Spherical Aberration (SA) by 25 % and with induced SA+coma by 61 % on average (3.0-mm pupils). DOF was 2.50 D in virgin eyes with EDOF IOL, 1.66±0.30 and 2.54±0.31 D (p<0.05) on average in post-LASIK eyes for 3.0-mm pupils, monofocal and EDOF IOLs, respectively. Halos were more sensitive to SA induction for 5.0-mm pupil and induction of positive SA (myopic LASIK) resulted in reduced halos with the EDOF when compared with the monofocal IOLs, by 1.62 (SA) and 1.86 arc min (SA+coma), on average.

**Conclusions:**

Computer post-LASIK pseudophakic eye models showed that DOF was less dependent on the presence of SA and coma with EDOF IOL and that halos were reduced with EDOF IOL compared to the monofocal IOL for a range of SA.

## Introduction

LASIK (laser-assisted in-situ keratomileusis) is a well-known ocular treatment, with more than 30 years of evolution^1^, which reshapes the cornea for the correction of refractive errors. Typically, an excimer laser is used to ablate the cornea, following an ablation profile such that the central cornea is flattened in myopic LASIK or steepened with respect to the periphery in hyperopic LASIK. While the theoretical Munnerlyn algorithm does not necessarily induce high order aberrations^2^, approximations of this algorithm, and discrepancies due to laser efficiency losses on a curved surface, and ablation plume shielding caused induction of spherical aberration and coma, especially with larger corrections^3–7^.

Several studies in the early 2000s^8–11^ reported a change in corneal asphericity, and an associated increase in the magnitude of spherical aberration (SA) following LASIK. In particular, two works by our group reported induction of positive spherical aberration following myopic LASIK^3^ and induction of negative SA following hyperopic LASIK^4^. The induction of corneal SA in the anterior surface of the cornea was proportional to the magnitude of the spherical correction and was obtained from the data obtained in patients operated with the Bausch and Lomb, Technolas 217-C laser (wavelength 193nm; pulse repetition rate 50Hz and a peak radiant exposure of 120mJ/cm^2^)^3,4^. The studies also showed increased coma, although its magnitude was not statistically significantly correlated with the pre-operative spherical error^5^. In later studies, we showed through both computational simulations of the ablation process^6^ and experimental measurements^7^ that a large percentage of the induction of SA could be explained by the ablation efficiency loss in the periphery due to the non-normal incidence.

Many patients that underwent LASIK surgery in the 2000’s are in need of cataract surgery today. The presence of an early LASIK surgery has posed challenges in the application of the standard formulas of calculation of the IOL power calculation, due to the unusual corneal shape^12^. Furthermore, with an increasing number of IOL designs now available for cataract and presbyopia correction, the selection is even more complex^13^. Among the new IOLs, EDOF IOLs^14^, which generally exhibit refractive profiles, appear to be less prompt to image degradation and artifacts than diffractive IOLs, while they are designed to expand visual functionality at intermediate distances. A question of clinical value is to investigate whether these lenses may be well-suited to patients with unusually high amounts of high order aberrations, such as those following LASIK surgery.

In this study, we performed computational simulations in post-LASIK eye models virtually implanted with the EDOF Acrysof IQ Vivity IOL (Alcon), and the monofocal Acrysof IQ IOL (Alcon) as a control. The Acrysof IQ Vivity is a wavefront shaping lens, designed with a patented X-Wave technology, which, according to the manufacturer, consists of a 2.20-mm wavefront shaping optics in the central part of the anterior surface to stretch and shift the wavefront avoiding light splitting^15^. This design extends the focal range rather than creating multiple focal points, with the intended goal of enhancing intermediate vision without compromising far vision.

## Methods

### Computational simulations

Pseudophakic computer eye models were designed in OpticStudio (Zemax, Kirkland, WA) modifying the Le Grand full theoretical model eye^16^ by aspherizing the anterior surface and placing the IOL 4.5-mm behind the posterior corneal surface. The post-LASIK anterior surface of the cornea was optimized to induce a change in power and spherical aberration and the central thickness was modified in myopic LASIK.

In the non-LASIK cornea, the radius of curvature of the anterior surface of the cornea was 7.8-mm and the asphericity (−0.117) of the anterior surface was obtained optimizing the conic constant so that the spherical aberration of the anterior surface of the cornea with a refractive index of 1.3771 was +0.28-μm for a 6.0-mm pupil diameter^17^. The posterior surface was a spherical surface (radius 6.5-mm) and the central thickness 0.55 mm.

We simulated three different post-myopic LASIK corneas with low, medium and high negative refractive corrections (−2.5, −4.5 and −7.5 D) and two post-hyperopic LASIK corneas with low and medium positive refractive corrections (+2.5 and +4.5 D). The SA induced by these corrections was obtained from the experimental data previously reported^4^ on the anterior corneal high order aberrations induced by LASIK surgery with a B&L, Chiron Technolas 217-C, equipped with the PlanoScan software: −0.223+0.169*SE μm/D for myopic LASIK and −0.050−0.277*SE mm/D for hyperopic LASIK for a 6.5-mm pupil diameter^4^, where SE stands for the pre-op spherical error corrected. The anterior surface radius of curvature and conic constant were modified to produce a change in power equal to the pre-op spherical error, and to induce spherical aberration. The conditions in the study^4^, single surface cornea with a 1.3391 refractive index and a 6.5-mm pupil diameter, were reproduced to induce the right amount of spherical aberration and the resulting conic constants were comparable to the ones reported experimentally^6^. Table 1 shows the SA induced in the different post-myopic and post-hyperopic LASIK tested conditions. Induction of coma (0.5-μm for a 6.5-mm pupil diameter, consistent with the reported amount)^5^ was also studied modeling the anterior surface of the cornea with a ZernikeSag surface type (an aspheric surface plus a surface described with Zernike polynomials) and modifying the coma term to obtain the desired change in the wavefront aberrations of the anterior surface. Corneal thickness in post-myopic LASIK cornea models was calculated using the Munnerlyn formula^2^ and an ablated zone of 6-mm^4^ which resulted in a loss of 12 μm per diopter. Corneal thickness in post-hyperopic LASIK cornea models was not modified (0.55-μm). In all eye models the shape of the posterior surface of the cornea was not altered (spherical surface, radius 6.5 mm), in agreement with our previous study that shows minimal changes after LASIK surgery^18^.

The virtually “implanted” IOLs were either the Acrysof IQ Vivity (EDOF IOL) or the Acrysof IQ (control monofocal IOL), with 22 D labeled power. According to the technical product information^15^, both IOLs were made of the same material (acrylate/methacrylate copolymer), exhibit anterior aspheric biconvex shape, have the same optical zone diameter (6.0-mm), overall length (13-mm), haptic angle (0 degrees) and UV blue light filtering. The IOL surface geometry, IOL thickness and refractive index at 555-nm were provided by the manufacturer. The axial length of the computer model eye was adjusted such that far images projected on the retina were in focus, with minimum spot radial size.

### Optical quality and Depth of focus.

**Figure 1:**
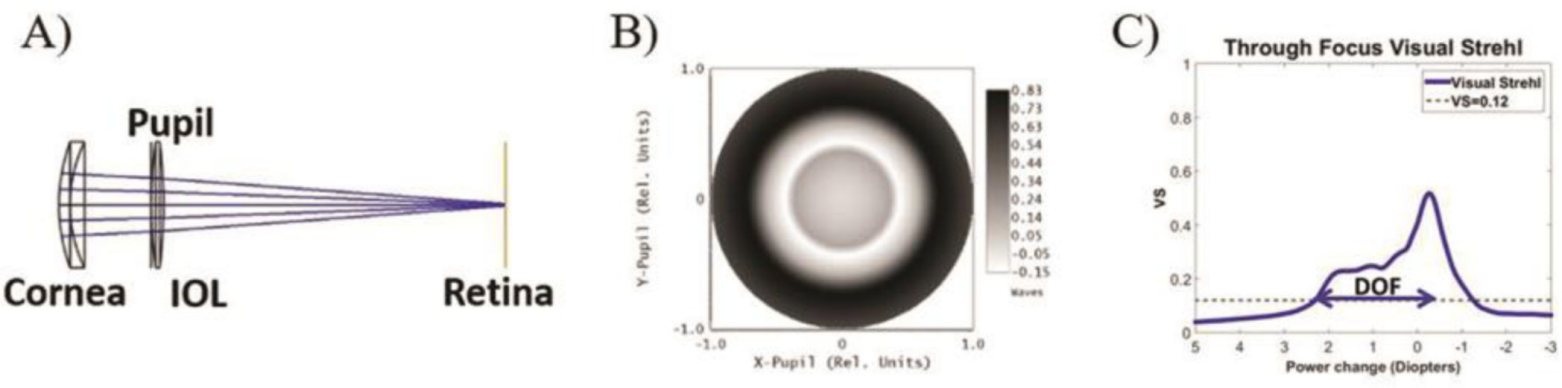
Illustration of the study methodology: A) representation of a computational pseudo-phakic eye model in Zemax. B) Wavefront aberration calculated from the computational eye model. C) Though Focus Visual Strehl (TFVS) curve, calculated from the corresponding wavefront at 3.0-mm pupil diameter. DOF is defined as the dioptric range for which VS is above 0.12 using the positive diopter range (blue horizontal dashed line).

The eye’s wavefront aberrations were obtained by ray tracing with OpticStudio using a mesh of 256×256 points for 3.0 and 5.0-mm pupil diameters (Fig 1-A and B). The Point Spread Function (PSF) and Modulation Transfer Function (MTF) were calculated using standard Fourier Optics-based routines written in MATLAB (MathWorks, Natik, MA), in an 8-D focus range, in 0.1-D steps. Retinal image quality was described in terms of Visual Strehl (VS) which is calculated from the wave aberration as the relative volume under the MTF (normalized to that of a diffraction limited system) weighting the frequency components with the average neural contrast sensitivity function. VS has been shown to correlate with visual performance^19–21^, and is often used to compare performance of different vision correction alternatives.

The following metrics were analyzed as a function of LASIK induced SA and coma: VS at far (0D) and depth of focus (DOF), defined as the usable defocus range for which VS>0.12^21^ (represented with the dashed horizontal blue line in Fig 1-C). These metrics were calculated in all conditions and used to compare the performance of eyes (virgin and post-LASIK) virtually implanted with either the EDOF or the monofocal IOL.

### Computational simulation of halos.

**Figure 2:**
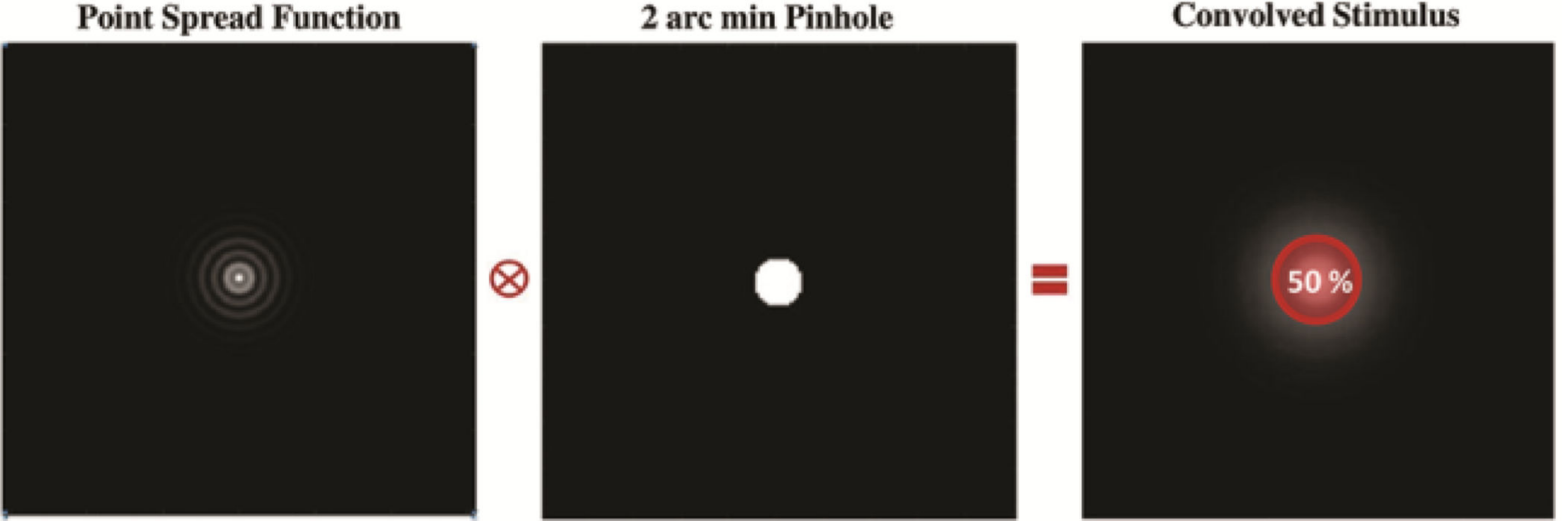
Methodology used to simulate halos: The retinal image was simulated by convolving the PSF with a 2-arcmin pinhole and the diameter that encircles 50% of the energy was calculated.

The presence of halos was estimated with a method similar to that described by Alba-Bueno et al.^22^ Retinal images of a 2-arcmin pinhole stimulus were simulated by convolution with the PSF of the eye, and the diameter that encircles 50% of the intensity was calculated (illustration in figure 2). The higher values of this metric, the more spread the halos in the image.

## Results

**Figure 3:**
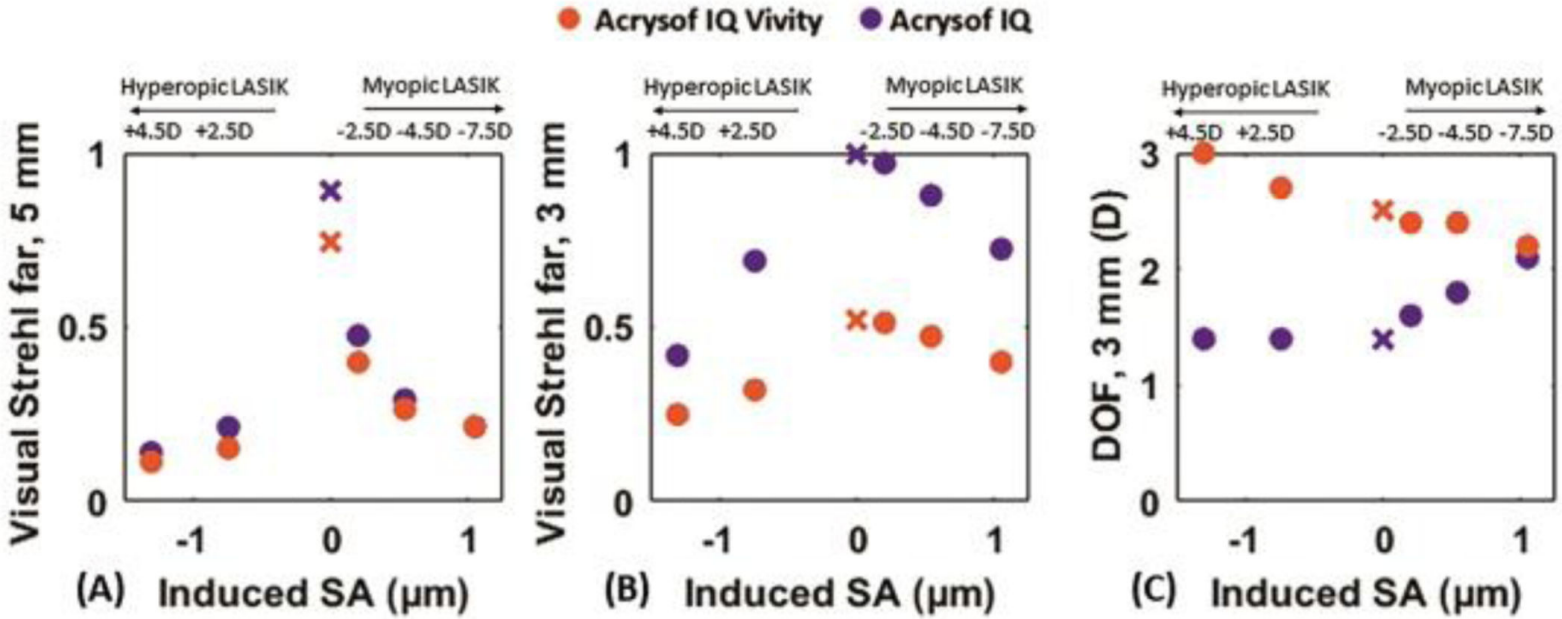
(A) VS for 5-mm pupil diameter; (B) VS for 3-mm pupil diameter; and (C) DOF for 3-mm pupil diameter as a function of induced SA (values for 6.5-mm pupil diameter) for the eye model “implanted” with the Acrysof IQ Vivity IOL (orange) and Acrysof IQ IOL (purple). The cross-shaped marker represents the VS value of a pseudophakic eye with an average virgin (non-surgical) cornea implanted with the Acrysof IQ Vivity IOL (orange) and with Acrysof IQ IOL (purple).

Figure 3 shows the estimated VS at far for 5-mm (left) and 3-mm pupil diameter (center) and the associated depth-of-focus for 3-mm pupil diameter (right) in the pseudophakic eye models, as a function of induced SA, for eyes implanted with the EDOF IOL (orange circles) and the monofocal IOL (purple circles) and the value for virgin eyes without LASIK treatment (orange and purple crosses).

The VS at far in the eye model with EDOF and monofocal IOL for 5-mm pupils in virgin eyes is 0.89 and 0.74 for monofocal and EDOF IOL, respectively, and differed by 0.04 on average across SA (Fig. 3-A). Only for 0 and 0.2-μm of induced SA, VS in the eye with the monofocal IOL exceeded notably (by 0.15 and 0.06, respectively) that of the eye with the EDOF IOL. The presence of SA induced by LASIK produced a sharp decrease in VS at far in post-myopic and post-hyperopic LASIK eyes from values >0.75 in non-LASIK eyes to <0.3 in eyes with ±1-μm induced SA (over 6.5-mm pupil) for both IOLs.

The VS at far for 3-mm (Fig. 3-B) exhibits a very different performance from that for 5-mm, 0.99 and 0.52 for virgin eyes with monofocal and EDOF IOL, respectively. The average VS was on average 0.37 higher with the monofocal than with the EDOF IOL. Also, for 3-mm the decrease in VS in post-LASIK eye models compared to virgin eyes was larger when negative spherical aberration was induced (i.e. in post-hyperopic LASIK model eyes).

DOF for 3-mm pupils (Fig 3-C) with the EDOF IOL was higher in corneas with induced negative SA (post-hyperopic LASIK) while DOF with the monofocal IOLs was higher in corneas with induced positive SA (post-myopic LASIK). The rate of change of the DOF with the EDOF IOL in the post-myopic LASIK eye models was slower than with the monofocal (−0.04 μm/D for EDOF IOL and 0.09 μm/D for monofocal IOL). In virgin eyes, DOF was 2.50 D with the EDOF IOL and 1.40 D with the monofocal IOL and the mean DOF difference between EDOF and monofocal IOL was 1.45 D in post-hyperopic LASIK eyes, and 0.50 D in post-myopic LASIK eyes. On average across conditions, DOF (mean±STD) was 2.53±0.28 and 1.62±0.29 D in post-LASIK eyes for 3.0-mm pupils, with EDOF and monofocal IOL, respectively and the difference between distributions was statistically significant (p<0.05).

**Figure 4:**
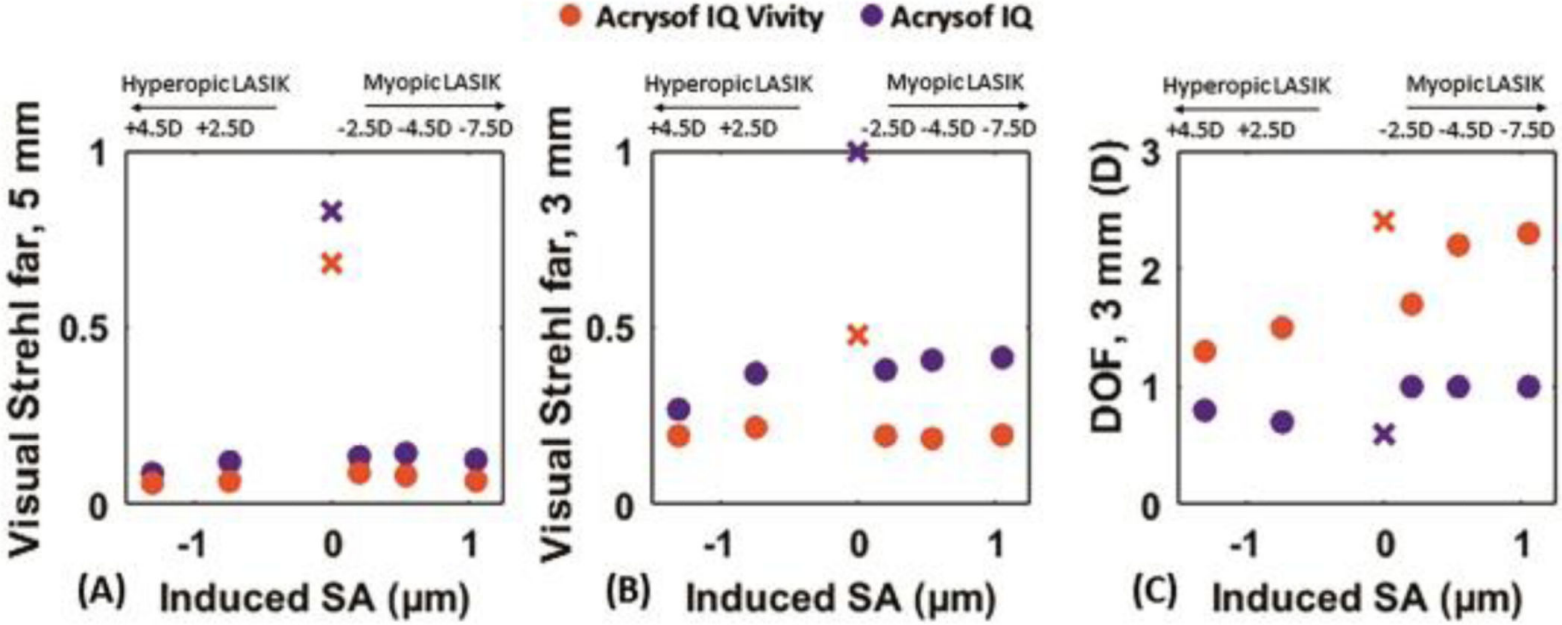
(A) VS metric for 5-mm pupil diameter; (B) VS for 3-mm pupil diameter; and (C) DOF for 3-mm pupil diameter as a function of induced SA for the eye model with Acrysof IQ Vivity IOL (orange) and Acrysof IQ IOL (purple) when both SA and coma are induced by LASIK. The cross-shaped marker shows the VS value of a pseudophakic eye with an average virgin (non-surgical) cornea implanted with the Acrysof IQ Vivity IOL (orange) and with Acrysof IQ IOL (purple).

Figure 4 shows similar analysis as Fig 3, but simulating SA in combination with 0.5-μm of coma induced by LASIK (at 6.5-mm pupil diameter). The results parallel those of the condition where only SA was induced, although the VS (for 5-mm pupils) further decreases by 0.14 on average, for both the EDOF and the monofocal IOL. For 3-mm pupil diameter VS decreased by 59 and 63% on average for EDOF and monofocal IOLs with respect to virgin eyes when SA and coma was induced.

**Figure 5:**
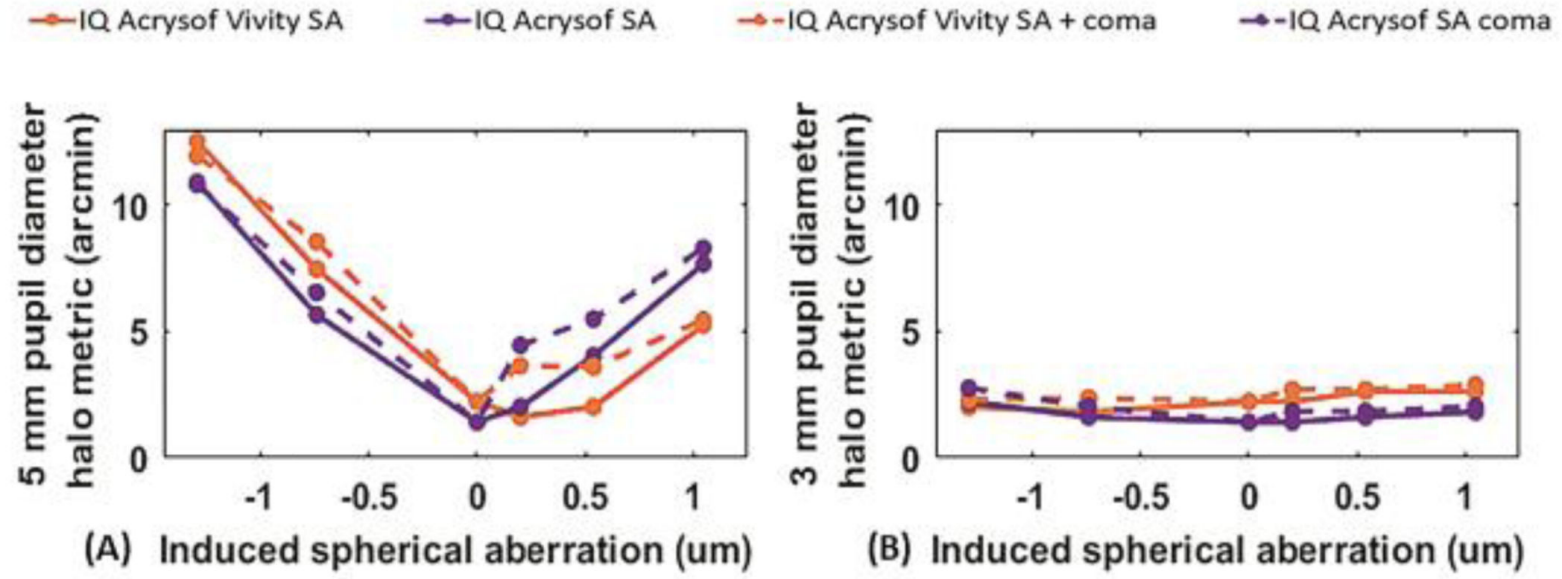
Halo metric in arcmin for EDOF and monofocal IOL as a function of LASIK-induced SA for a 5-mm pupil diameter (A) and 3-mm pupil diameter (B). The no treated cornea case is represented with 0-μm induction of SA. The continuous line represents the cases where only SA is induced, and the dashed line represents the combination of induced SA and coma.

Figure 5 shows the halo metric which accounts for the spatial size of halos (angular diameter, in arcmin, that encircles 50% of the energy), for 5-mm (Fig. 5-A) and 3-mm (Fig. 5-B) pupil diameters, for the EDOF IOL (orange) and the monofocal IOL (purple), for virgin corneas (zero induced SA), LASIK-induced SA (solid line), and LASIK-induced SA in combination with coma (dashed line).

With non-LASIK corneas, the halo metric was 1.41 arcmin for the monofocal IOL, and 2.22 arcmin for the EDOF IOL, for both 3 and 5-mm pupils. While the halo metric was fairly constant for 3-mm pupils, there was a sharp increase when increasing the magnitude of the induced SA for 5-mm pupils.

For 5-mm pupils, the halo metric with EDOF IOLs exceeded that obtained with monofocal IOLs when negative SA was induced (10.00 vs 8.29 arcmin on average for the EDOF and monofocal IOLs respectively when negative SA was induced). However, positive SA induced in myopic LASIK, resulted in reduced halos with the EDOF when compared with the monofocal IOLs, by 1.6 (SA) and 1.9 (SA+coma) arcmin, on average. For 3-mm pupil diameters, the presence of coma had a minimal impact with an average increase across conditions of 0.27 and 0.29 arcmin for EDOF and monofocal IOL respectively.

## Discussion

We evaluated the performance of two intraocular lenses using computer model eyes with post-LASIK corneas, simulated adding the SA or SA and coma that was induced during surgery in the early 2000s.^3–5^ The studied IOLs were a monofocal aspheric IOL, Acrysof IQ, and the EDOF IOL Acrysof Vivity IQ, by Alcon.

Our computer eye model follows standards proposed in the literature^23^. In particular, we used a (non-surgical) corneal model that mimics the ISO standard, both in power (43 D) and magnitude of SA (0.28-μm, for a 6.0-mm diameter pupil)^17^. While this choice is slightly different to that followed by other studies in the literature^24–26^ that use the cornea defined in the Navarro eye model^27^ (42.16 D and +0.139 μm SA, 6-mm pupil), it is closer to the average population^17,^ and to on-bench testing parameters. To evaluate the through focus performance we used VS. An alternative proposed metric VS_combined_^28,29^ that considers the effects of phase on visual quality was also evaluated. The average difference (across patients and conditions) between VS and VS_combined_ was 0.09, and the relative through focus image quality performance was similar across metrics, so the more widely used VS was used for reporting.

An on-bench study^30^ compared the performance of Acrysof IQ Vivity with a trifocal IOL in terms of the MTF at 50 lp/mm and Strehl ratio for far objects using the ISO standard with corneal SA. These experimental results with Vivity IOL show a higher image quality for larger pupils than for smaller pupils at far, in good agreement with the results of our simulation. The study did not report DOF. A clinical study on 40 patients^31^, and another on 16 patients^32^ who were implanted binocularly with the Acrysof IQ Vivity IOL showed good far and intermediate vision and low reports of patients bothered by glare, halos or starburst.

Our comparisons between the simulated performance with the Acrysof IQ and Acrysof IQ Vivity IOLs in non-LASIK eyes can be contrasted with two studies that compared the clinical outcomes of these two lenses^33,34^. After 6 months of implantation, in photopic conditions, McCabe et al.^33,34^ found that the DOF was higher in the eyes implanted with the EDOF IOLs than the ones implanted with the monofocal. In addition, VA at 66-cm was higher for the EDOF IOL, in agreement with our findings for 3-mm pupil diameters. Additionally, data on the influence of pupil diameter in the performance of the Vivity IQ IOL described in an FDA report^35^ showed an increase in DOF in patients when the pupil diameter decreased, In fact, we found the same trend (increase in DOF from 1.40 to 2.50 D), when changing the pupil diameter from 5 to 3-mm. Furthermore, a study by Pastor-Pascual et al.^36^ presented aberrometry measurements on normal patients implanted with the monofocal IOL and with the EDOF IOL studied here. Their reported DOF estimates are based on VS calculations, and therefore directly comparable with our simulations which use the same metric. They show a high degree of correspondence with the current study: DOF with the EDOF IOL in the clinical study was 2.50D, 1.25 D broader than with the monofocal IOL (in our simulations 2.50 D, 1.10 D broader than with the monofocal, for 3.0-mm pupil diameters). These VS-based DOF values are also in close agreement with Gundersen et al.^31^, who reported a DOF obtained from binocular VA defocus curves of 2.50D in 40 patients implanted with EDOF IOL in a modified monovision strategy.

While to our knowledge there are no reports of clinical performance of the Vivity IOL in LASIK patients, the good correspondence between our computer simulations with clinical performance suggests that we can extrapolate a similar methodology to predict performance in post-LASIK patients. To describe post-operative corneas, we used aberrometry data in pre- and post-myopic and hyperopic patients obtained in our laboratory in the early 2000’s.^3–5^ While conclusions are limited to the aberrations induced by the particular laser used in these surgeries (B&L Technolas 217-C), and on the laser parameters such as the programmed algorithm and laser fluence^7^, the timeline appears realistic, as a number of patients who had LASIK 20 years ago are approaching presbyopic/cataract surgery today and it is still challenging today to choose a multifocal IOL in patients with reshaped corneas.^37–39^ A simplification of our approach is to represent the post-operative corneal surfaces with aspheric surfaces, instead of directly using the post-operative topography. However, Cano et al.^6^ in the same data set used in this study, found that the increase in asphericity was the main driver of the spherical aberration increase. We also studied the possibility of simulating the induced aberrations in the pupil plane (using OpticStudio ZernikePhasePlate surface type) as a phase-plate, instead of redesigning the corneal surface. We found that using this simplified approach the average differences with results using the full cornea were less than 0.1 for VS, and less than 0.3 D for the estimated DOF. However, we opted for the model presented here where the corneal anterior radius of curvature and the conic constant are modified to account for the change in power and spherical aberration. Another simplification is to study only one IOL power instead of the one that would be needed for the actual axial length of the individual subjects, or to consider a constant anterior chamber depth. We studied the influence of the anterior chamber depth in the eye spherical aberration and comma and found that in the range 3.2 to 4.5 mm, the change in the SA was 0.06 μm, which is much smaller than the SA induced by the cornea or the IOL under study.

Our simulations show significant degradation of the optical quality in post-LASIK eyes (circles in Figures 3 and 4) in comparison to non-surgical eyes (cross-markers in those figures), particularly for 5-mm pupil diameters, as higher amounts of aberrations with larger pupils degrade retinal image quality further. For small amounts of induced SA, the impact of LASIK on performance with implanted IOLs is markedly different in myopic and hyperopic LASIK (see asymmetries in the VS curves in Fig 3 (A)). This may be explained by the fact that negative SA induced by hyperopic LASIK cooperates with the negative SA of the aspheric IOL to compensate the positive corneal SA and because of the larger SA induced in hyperopic LASIK. For 5.0-mm pupil diameters this results in lower DOF with induced negative SA, with both the monofocal and EDOF IOLs.

While for 5-mm pupils both the monofocal and EDOF IOLs appear to be similarly impacted by the LASIK-induced aberrations, and the visual benefit of the EDOF IOL at intermediate distances is only slightly apparent in post-hyperopic LASIK corneas, the performance of the EDOF and monofocal IOLs is drastically different at 3-mm pupil diameters: visual degradation at far, visual benefit at near and DOF is consistently higher with EDOF IOLs in all conditions. Furthermore, at 3-mm pupils, the presbyopic-correction profile of the EDOF IOL appears to prevail above the LASIK-induced aberrations, such that the DOF is higher with the EDOF than with the monofocal IOL for the same cornea (except for the highest myopic LASIK eye) and remains relatively constant regardless the magnitude of corneal aberrations. For 5-mm both IOLs produce similar DOF, 1.13±0.41D and 1.14±0.40D on average for monofocal and EDOF IOL respectively.

Halos were quantified by the diameter encircling 50% of the energy. Computational and on bench studies on the halos produced by multifocal IOLs^22^, showed that halos depend on IOL addition, design, and pupil diameter. In post-LASIK eyes, halos are a direct consequence of the induced SA and coma. Unlike other EDOF IOLs, the principles of operation of the EDOF IOL under study do not rely on manipulating the spherical aberration, and therefore spherical aberration and halos do not appear to be coupled when implanted in post-LASIK corneas. As expected, our simulations on non-surgical corneas show slightly larger halos with the EDOF IOL than the monofocal IOL (cross markers in Fig 5), and a very small impact of pupil diameter on halo size (cross markers in Fig 5-(A) vs Fig 5(B)). These differences do not appear to be clinically relevant, as recently published in questionnaire-based reports^40^. The larger halos in post-LASIK eyes for 5-mm than for 3-mm pupils indicate a larger contribution of the LASIK-induced aberrations on halos. Post-hyperopic LASIK eyes with monofocal IOLs exhibit smaller halos than post-myopic LASIK eyes (5.0-mm pupils), presumably because of the above-mentioned compensatory effect of SA. In the presence of coma, the value of the halo metric increased more symmetrically with positive and negative SA, suggesting a larger influence of coma over SA in the halo extension.

Interestingly, the EDOF IOL appears to protect against the halos produced by myopic LASIK, resulting (for the same post-LASIK cornea) in significantly smaller halos than with monofocal IOL when positive SA is induced at 5-mm pupil diameter. While the nature of this favorable interaction between the EDOF IOL profile and SA needs further investigation, the finding is reminiscent of prior reports of favorable interaction between scattering and SA^41^. That study reported on bench and in vivo contrast sensitivity (CSF) measurements through diffusers, positive SA and various amounts of defocus, and found that, under several conditions, the combination resulted in higher contrast, concluding that the effect was of optical origin.

In conclusion, we have built models of post-LASIK pseudophakic eyes using published data on the LASIK-induced SA and coma, and geometrical information of the IOLs (monofocal and EDOF IOLs). Our simulations show that the Acrysof IQ Vivity IOL produces a significant benefit at intermediate distance at the expense of some degradation at far, although those differences are unlikely clinically relevant. For large pupils the Vivity IOL behaves similarly to a monofocal IOL, but significantly enlarges the DOF for smaller pupils. In post-LASIK eyes, performance of the Acrysof IQ Vivity is rather immune to the presence of high order aberrations, and exhibits a quite constant DOF for a large range of corneal positive or SA. The Vivity IOL appears particularly suitable in post-myopic LASIK surgery eyes, given the larger DOF expected compared to that produced by the monofocal IOL for smaller pupils, and the smaller halo for larger pupils. While our computer eye models capture corneal shape and the IOL geometry, several aspects could make them rather realistic. For example, computer eye models can incorporate patient-specific geometrical and biometric data of the cornea and lens, IOL tilt and decentrations, pupil decentrations, or the off-axis location of the fovea, which have shown to reproduce the eye’s wave aberration with great accuracy.^42,43^ Also, previous studies from our group have reported on the effect of manufacturing variability and centration experimentally with another IOL^44^, which could be the object of a future study. Further support to these recommendations could be achieved through visual simulations in real post-LASIK patients. On bench Adaptive Optics Visual Simulators^45,46^ or wearable visual simulators (SimVis)^47^ are capable of simulating IOLs by mapping a phase map representing the IOLs or by temporal multiplexing^46,48–50^. Understanding the coupling of the IOL design with the corneal aberrations and pupil size through computer and visual simulations is a valuable avenue to improve lens design and customized IOL selection.

## Supplementary Material

additional references

## Figures and Tables

**Table 1. -:** Corneal spherical aberrations induced with LASIK surgery for different preoperative spherical errors at 6.5 mm pupil diameter

Preop spherical error (D)	Induced spherical aberration (μm) 6.5 mm pupil diameter
High myopia, −7.5 D	+1.045
Mid myopia, −4.5 D	+0.538
Low myopia, −2.5 D	+0.200
Low hyperopia, +2.5 D	−0.743
Mid hyperopia, +4.5 D	−1.297

## Value statement

### What was known

- Several studies in the early 2000s reported a change in corneal asphericity, and an associated increase in the magnitude of spherical aberration after LASIK surgery proportional to the spherical magnitude of spherical correction.
- Some studies also showed increased coma after LASIK surgery and suggested that it could be originated by the decentration of the ablation profile due to the off axis foveal position.
- Many patients that had undergone LASIK surgery in the 2000’s are in need of cataract surgery today. The presence of an early LASIK surgery has posed challenges in the application of the standard formulas of calculation of the IOL power calculation, due to the unusual corneal shape.

### What this paper Adds

- Our simulations show that, when compared with a monofocal IOL, the Acrysof IQ Vivity IOL produces a larger DOF (1.4 and 2.5 D for monofocal and Vivity IOLs, respectively) at the expense of some degradation at far (VS 0.99 and 0.52 for monofocal and Vivity, respectively), for 3-mm pupils.
- In post-LASIK eye models, the DOF with Acrysof IQ Vivity is rather immune to the presence of high order aberrations, and exhibits a quite constant value (less than 1.0 D change) for a large range of corneal SA.
- While the halo metric was fairly constant for 3-mm pupils, there was a sharp increase in halo size when increasing the magnitude of the induced SA for 5-mm pupils. Halos with EDOF IOL when positive SA was induced, i.e. with myopic LASIK, were smaller than the ones produced with monofocal IOL.
