# Supplementary material for: Computational simulation of the optical performance of an extended depth of focus intraocular lens in post-LASIK eyes
Source: J Cataract Refract Surg. Author manuscript; Available in PMC 2025 May 14. (PMC12077381; doi:10.1097/j.jcrs.0000000000001260)
Supplement: additional references [file NIHMS1998561-supplement-additional_references.docx]

1. Pastor-Pascual F, Gómez-Gómez A, Montés-Micó R, Ruiz-Mesa R, Tañá-Rivero P. Polychromatic through-focus image quality in a wavefront-shaping presbyopia correcting intraocular lens. *Expert Review of Ophthalmology*. 2022;17(1):75-79. doi:10.1080/17469899.2022.2021878
2. Fisher B, Potvin R. Clinical outcomes with distance-dominant multifocal and monofocal intraocular lenses in post-LASIK cataract surgery planned using an intraoperative aberrometer. *Clinical & Experimental Ophthalmology*. 2018;46(6):630-636. doi:10.1111/ceo.13153
3. Naseri A, McLeod SD. Cataract surgery after refractive surgery. *Curr Opin Ophthalmol*. 2010;21(1):35-38. doi:10.1097/ICU.0b013e328333e9ab
4. Iijima K, Kamiya K, Shimizu K, Igarashi A, Komatsu M. Demographics of patients having cataract surgery after laser in situ keratomileusis. *J Cataract Refract Surg*. 2015;41(2):334-338. doi:10.1016/j.jcrs.2014.05.045
5. Rementería-Capelo LA, Lorente P, Carrillo V, Sánchez-Pina JM, Ruiz-Alcocer J, Contreras I. Patient Satisfaction and Visual Performance in Patients with Ocular Pathology after Bilateral Implantation of a New Extended Depth of Focus Intraocular Lens. *Journal of Ophthalmology*. 2022;2022:e4659309. doi:10.1155/2022/4659309
6. Pérez GM, Manzanera S, Artal P. Impact of scattering and spherical aberration in contrast sensitivity. *Journal of Vision*. 2009;9(3):19. doi:10.1167/9.3.19
7. Rosales P, Marcos S. Customized computer models of eyes with intraocular lenses. *Opt Express*. 2007;15(5):2204-2218.
8. Tabernero J, Piers P, Benito A, Redondo M, Artal P. Predicting the optical performance of eyes implanted with IOLs to correct spherical aberration. *Invest Ophthalmol Vis Sci*. 2006;47(10):4651-4658. doi:10.1167/iovs.06-0444
9. Pérez-Merino P, Marcos S. Effect of intraocular lens decentration on image quality tested in a custom model eye. *Journal of Cataract & Refractive Surgery*. 2018;44(7):889-896. doi:https://doi.org/10.1016/j.jcrs.2018.02.025
10. Fernández EJ, Manzanera S, Piers P, Artal P. Adaptive Optics Visual Simulator. *J Refract Surg*. 2002;18(5):S634-S638. doi:10.3928/1081-597X-20020901-27
11. Vinas M, Aissati S, Romero M, Benedi-Garcia C, Garzon N, Poyales F, Dorronsoro C, Marcos S. Pre-operative simulation of post-operative multifocal vision. *Biomed Opt Express, BOE*. 2019;10(11):5801-5817. doi:10.1364/BOE.10.005801
12. Marcos S, Vinas M, Dorronsoro C, Sawides L, Gambra E, Benedí C, Elaissati S. Adaptive-Optics based visual simulators: from on-bench to wearable devices. In: OSA Technical Digest. Optical Society of America; 2018:OTh4C.2. doi:10.1364/AOMS.2018.OTh4C.2
13. Akondi V, Dorronsoro C, Gambra E, Marcos S. Temporal multiplexing to simulate multifocal intraocular lenses: theoretical considerations. *Biomed Opt Express*. 2017;8(7):3410-3425. doi:10.1364/BOE.8.003410
14. Vinas M, Benedi-Garcia C, Aissati S, Pascual D, Akondi V, Dorronsoro C, Marcos S. Visual simulators replicate vision with multifocal lenses. *Sci Rep*. 2019;9(1):1539. doi:10.1038/s41598-019-38673-w
15. Radhakrishnan A, Pascual D, Marcos S, Dorronsoro C. Vision with different presbyopia corrections simulated with a portable binocular visual simulator. *PLoS One*. 2019;14(8):e0221144. doi:10.1371/journal.pone.0221144
